# The Effects of Prioritizing Lead or Boulder Climbing Among Intermediate Climbers

**DOI:** 10.3389/fspor.2021.661167

**Published:** 2021-04-22

**Authors:** Nicolay Stien, Tor Frithjof Frøysaker, Espen Hermans, Vegard Albert Vereide, Vidar Andersen, Atle Hole Saeterbakken

**Affiliations:** Department of Sport, Food and Natural Sciences, Institute of Education, Arts and Sports, Western Norway University of Applied Sciences, Sogndal, Norway

**Keywords:** strength, isometric, sport, training, physical performance

## Abstract

This study compared the effects of prioritizing lead climbing or boulder climbing on climbing-specific strength and endurance, as well as climbing performance. Fourteen active climbers were randomized to a boulder climbing training group (BCT: age = 27.2 ± 4.4 years, body mass = 65.8 ± 5.5 kg, height = 173.3 ± 3.8 cm) or a lead-climbing training group (LCT: age = 27.7 ± 6.1 years, body mass = 70.2 ± 4.4 kg, height = 177.7 ± 4.4 cm). The groups participated in a 5-week training period consisting of 15 sessions, performing either two weekly bouldering sessions and one maintenance-session of lead-climbing (BCT) or two weekly lead-climbing sessions and one maintenance-session of bouldering (LCT). Pre- and post-training, maximal force and rate of force development (RFD) were measured during isometric pull-ups performed on a jug hold and a shallow rung, and during an isolated finger-strength test. Lead-climbing and bouldering performance were also measured, along with an intermittent forearm endurance test. The pre-to-post changes were not significantly different between the groups for any of the parameters (*P* = 0.062–0.710). However, both the BCT (ES = 0.30, *P* = 0.049) and LCT (ES = 0.41, *P* = 0.046) groups improved strength in the isometric pull-up performed using the jug, whereas neither group improved force in the rung condition (*P* = 0.054 and *P* = 0.084) or RFD (*P* = 0.060 and *P* = 0.070). Furthermore, climbing and bouldering performance remained unchanged in both groups (*P* = 0.210–0.895). The LCT group improved forearm endurance (ES = 0.55, *P* = 0.007), while the BCT group improved isolated finger strength (ES = 0.35, *P* = 0.015). In addition to isometric pull-up strength, bouldering can increase isolated finger strength while lead-climbing may improve forearm endurance. A 5-week period prioritizing one discipline can be safely implemented for advanced to intermediate climbers without risking declined performance in the non-prioritized discipline.

## Introduction

In the last decades, rock climbing has become increasingly popular among athletes, recreational practitioners, and researchers. The most practiced disciplines of the sport are lead climbing and bouldering. Whereas boulder problems usually include 5–10 very powerful moves on short routes (<5 m) (White and Olsen, 2010), lead climbing is typically performed on higher walls and requires the climber to perform several moves on a sub-maximal intensity over a longer time (e.g., 2–7 min) (Mermier, 2000). Common for both climbing disciplines are high levels of mental, technical, and physiological demands (MacLeod et al., 2007; Draper et al., 2008; Magiera et al., 2013; Levernier and Laffaye, 2019a; Taylor et al., 2020). Since the sport consists of maintaining contact with the holds and generating vertical movement, strength and endurance of the finger flexors and the pulling apparatus (elbow-flexors and shoulder-extensors) are generally accepted as key-factors for climbing performance (MacLeod et al., 2007; Laffaye et al., 2016).

Researchers have examined different training interventions for improving the aforementioned factors among climbers. Fingerboard training is the most examined form of climbing-specific training in the scientific literature and has proven efficient for improving maximal finger strength and forearm endurance (López-Rivera and González-Badillo, 2012, 2019; Medernach et al., 2015; Levernier and Laffaye, 2019a; Philippe et al., 2019). Importantly, the improvements have been in climbing-specific tests (e.g., dead-hang endurance or dynamometer finger-grip strength) similar to the implemented fingerboard training (López-Rivera and González-Badillo, 2012, 2019; Levernier and Laffaye, 2019a), which, according to the principle of specificity (Sale and MacDougall, 1981), may not transfer directly to actual climbing performance. Moreover, the fingerboard training in the mentioned studies only involved isometric training and thereby lack specificity to the dynamic movement pattern in climbing. Improved climbing performance has yet to be demonstrated following improvement in climbing-specific tests. To the authors knowledge, only one study (Anderson and Anderson, 2015) has reported a correlation between improvements in finger strength and climbing performance. However, the training was unsupervised and the changes in strength and performance were self-reported, meaning the findings should be interpreted with caution.

Previous assessments of athletes specializing in either bouldering or lead climbing have found distinctive physiological differences between disciplines (Fanchini et al., 2013; Laffaye et al., 2014; Fryer et al., 2017; Stien et al., 2019; Levernier et al., 2020). Likely due to the different physiological demands of the two disciplines (White and Olsen, 2010), boulderers have performed better than lead climbers in isometric [maximal voluntary isometric contraction (MVIC) and rate of force development (RFD)] and dynamic tests (pull-up velocity and power output), with RFD being the most discriminatory factor (Fanchini et al., 2013; Laffaye et al., 2014; Stien et al., 2019). Conversely, no difference in forearm endurance or oxidative capacity has yet been detected between disciplines (Fryer et al., 2017; Stien et al., 2019). Cross-sectional studies are inherently unable to answer whether the differences between lead- and boulder climbers are physiological adaptations specific to the discipline, or a result of climbers choosing to engage the discipline best suited to their inherent physiological abilities. Currently, only one study (Philippe et al., 2019) has examined the effects of prioritizing lead or boulder climbing for 8 weeks among advanced climbers. The authors reported similar lead-climbing performance improvements in both groups. However, the bouldering group performed both disciplines (i.e., lead-climbing and bouldering), whereas the muscular endurance group only performed lead climbing, thereby possibly confounding the results.

Still, implementing a maintenance-session in which the non-prioritized discipline is performed with low intensity in both groups, could preserve specific qualities whilst emphasizing the prioritized discipline, as shown among cyclists and soccer players (Rønnestad et al., 2011, 2014). On the basis of the previously observed differences between athletes specializing in the two climbing disciplines and the scarce scientific literature on the effects on lead-climbing or bouldering, the aim of this study was to compare the adaptations to performing mainly lead climbing or boulder climbing for a 5-week period among intermediate and advanced climbers. Based on the previous findings (Fanchini et al., 2013; Fryer et al., 2017; Stien et al., 2019), it was hypothesized that the bouldering group would demonstrate superior improvements in RFD and MVIC during an isometric pull-up and an isolated finger-grip strength test, whereas the two groups would similarly improve forearm endurance measured in an intermittent test. Both groups were further expected to improve climbing performance more in their prioritized discipline than in their non-prioritized discipline, as well as to improve their prioritized discipline more than the other group.

## Materials and Methods

### Experimental Approach to the Problem

A randomized trial was designed to examine the effects of prioritizing either lead climbing or bouldering for 5-weeks. Before and after the intervention, subjects underwent the following tests: (1) maximal average force over 2 s (F_avg_) was measured while performing an isometric pull-up on a 23 mm rung, (2) F_avg_ and RFD were collected during an isometric pull-up using a jug hold, (3) isolated finger-grip strength was collected using a custom built apparatus, (4) forearm muscle endurance was measured using an intermittent finger flexion test to failure, (5) bouldering performance was assessed on three boulder problems, and (6) lead-climbing performance was tested on an 18 m indoor climbing wall. The order of the tests was standardized to avoid variations in fatigue.

### Subjects

With a statistical level set to 0.05, a statistical power of 80%, and using the 25–30% change in RFD in a comparable study (Levernier and Laffaye, 2019a), a total of 10 subjects (five in each group) was the minimum number of subjects required to detect a significant difference. Fourteen climbers (seven in each group) were recruited and completed the training and testing. All included subjects conducted both bouldering and lead climbing regularly and were not specialized in either discipline. Following pre-testing, subjects were randomly allocated to the lead climbing training group (LCT) or the boulder climbing training group (BCT) by drawing lots from a non-transparent container. See Table 1 for group characteristics. The inclusion criteria were a minimum self-reported red-point grade of 6b+ (IRCRA: 14) for women and 6c (IRCRA: 15) for men, as well as absence of injuries in the last 6 months. Subjects were informed verbally and in writing about the potential risks and benefits of participation and signed and informed consent form before data collection commenced. The research procedures were in accordance with the ethical guidelines of the university, approved by the Norwegian Center for Research Data (reference number: 252152), and conformed to the standards of treatment of human participants in research as outlined in the 5th Declaration of Helsinki.

**Table 1:** Baseline anthropometric characteristics, climbing experience, number of weekly climbing sessions, and best achieved red-point grade at pre-test for the two groups.

	**Lead climbing group (6 male and 1 female)**	**Bouldering group (5 male and 2 female)**	**Between-groups *p*-value**
Age (years)	28.7(6.1)	27.2(4.4)	0.896
Height (cm)	177.7(4.4)	173.3(3.8)	0.137
Body mass (kg)	70.2(4.4)	65.8(5.5)	0.099
Fat mass (%)	13.1(2.1)	14.5(3.2)	0.269
Muscle mass (%)	86.8(4.9)	85.4(3.7)	0.157
Experience (years)	7.7(6.9)	5.0(2.2)	0.105
Weekly sessions (*n*)	3.4(0.5)	3.3(0.7)	0.990
Best red-point (IRCRA)	17.5(1.9)	15.0(1.8)	0.112

*IRCRA = grade given using to the numerical grading system proposed by the International Rock Climbing Research Association*.

*Values are presented as means (±95% confidence intervals)*.

### Methodology

Upon arrival to the laboratory, subjects were interviewed about their climbing performance and weekly climbing volume before anthropometric variables (height and body mass) and body composition (fat percentage and muscle mass) were measured using a wall mounted measuring tape and a bioelectric impedance weight (Tanita MC780MAS, Tokyo, Japan), respectively. Following the interview and anthropometric measurements, a 15-min light-to-moderate warm-up consisting of bouldering and traversing was performed. The subjects selected the difficulty of the boulders themselves but were instructed to avoid fatigue. The warm-up and testing were separated by 5 min of passive rest.

**Figure 1:**
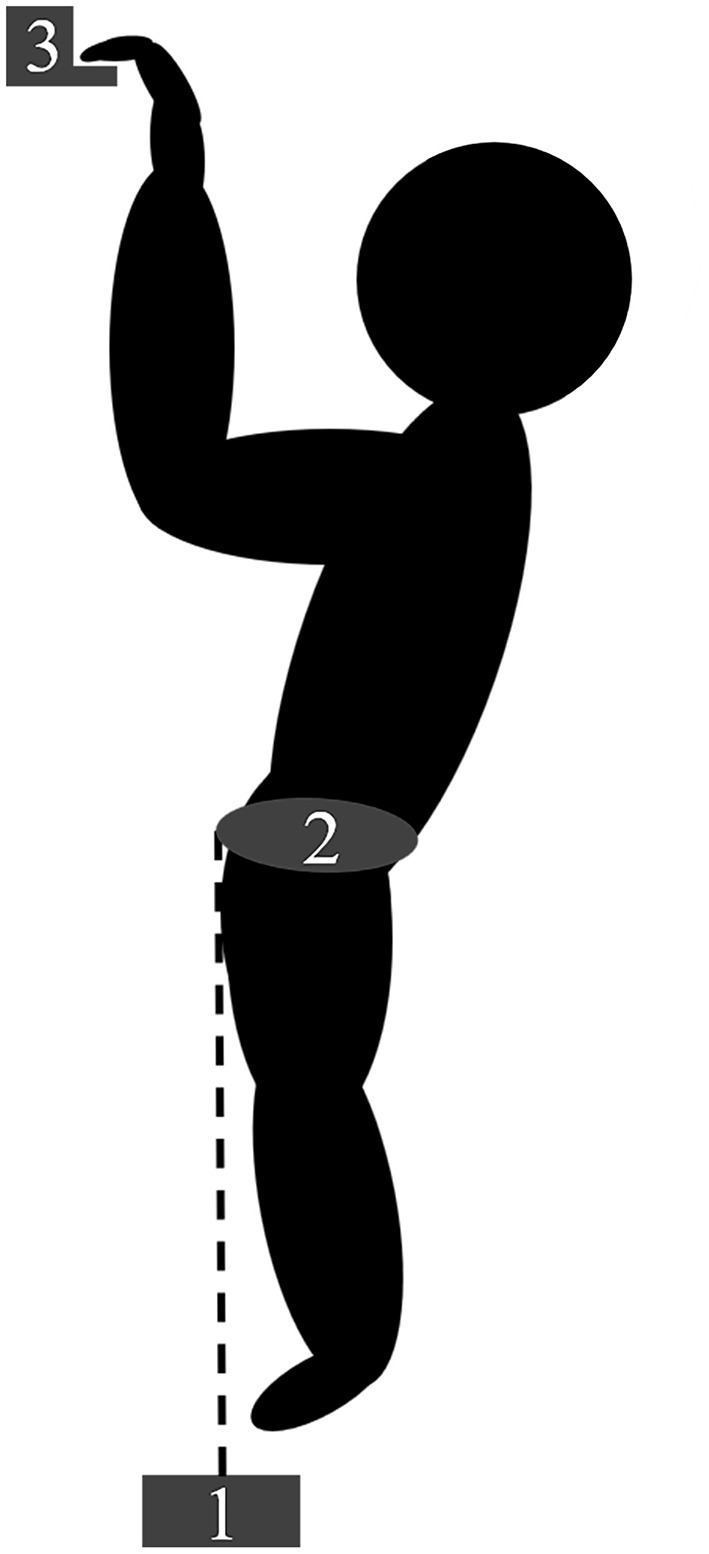
Schematic presentation of the maximal pull-up strength test set-up showing (1) the force cell, (2) the climbing harness, and (3) the hold used (the rung condition is presented, but the set-up was identical for the jug condition).

The subjects performed two conditions (rung and jug holds) of three maximal voluntary isometric pull-ups, each separated by 3 min rest intervals. All tests were conducted using both hands. In the rung condition, a 23 mm deep rung with rounded edges was used (Metolius Climbing, Bend, Oregon, USA) with a half crimp grip and a passive thumb. This test intends to measure the maximal, isometric pull-up strength in a climbing-specific condition where finger strength is the limiting factor (Stien et al., 2019). For the jug condition, a Beastmaker 1000 Series (Beastmaker Limited, Leicester, United Kingdom) was used. This test allows the subjects to fully engage the pulling apparatus without being limited by finger strength. The subjects wore a climbing harness anchored to the ground *via* an expansion bolt, a static daisy-chain and a force cell with 200 Hz resolution (Ergotest Innovation A/S, Porsgrunn, Norway; Figure 1). The force cell was regularly calibrated using a 20 kg weight. The force was registered using a computer with the commercial software MuscleLab (v.10.4.37.4073, Ergotest Innovation A/S, Porsgrunn, Norway). The daisy chain was adjusted to allow each subject to remain in a 90° elbow angle (measured with goniometer).

**Figure 2:**
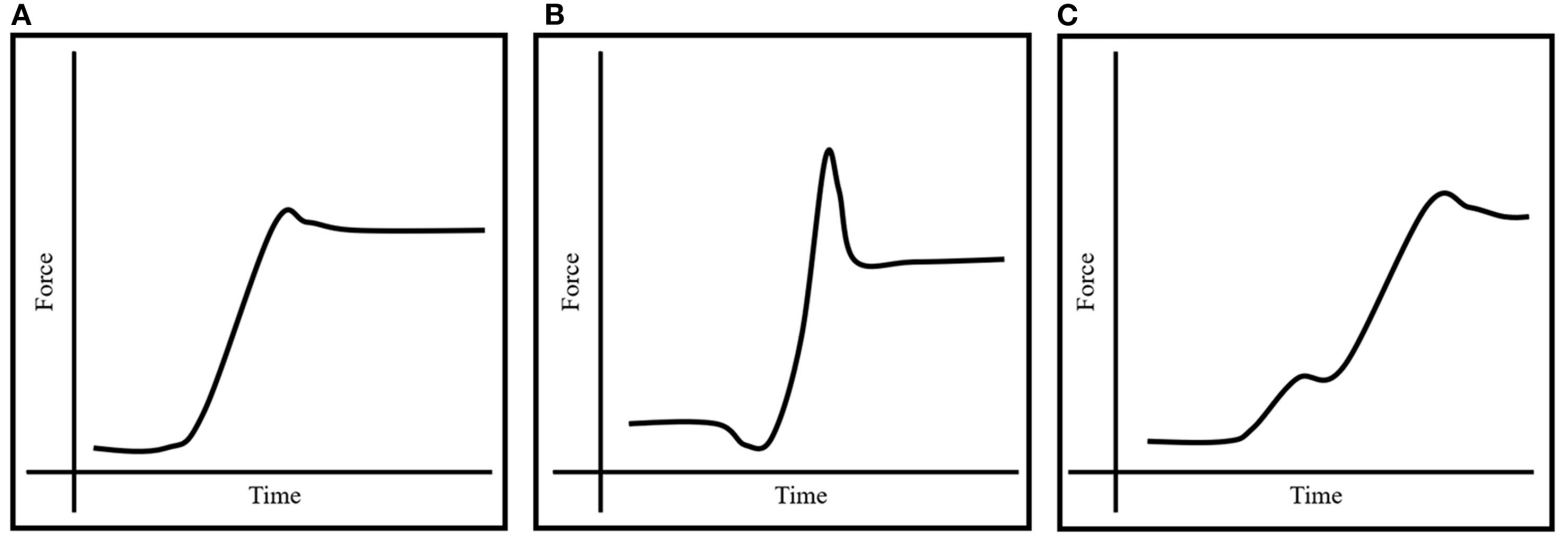
Illustrations showing an acceptable force curve **(A)**, a curve produced while chipping the legs to create upward momentum **(B)**, and a curve in which the force plateaus before reaching peak force **(C)**.

On verbal command, the subjects pulled themselves up to a 90° elbow angle (where the daisy-chain became taut) and remained in that position 1–3 s to create a stable baseline (no more than ±5 N fluctuations in baseline force) before being instructed to perform an isometric pull-up as fast and as hard as possible and maintain the force output for ~3 s (Figure 2A). An attempt was annulled if any chipping of the legs was used to create upward momentum (Figure 2B), or if the force output plateaued before reaching peak force (Figure 2C). The F_avg_ was extracted from the 2-s window with the highest average force output. The F_avg_ in the best attempt on the rung [coefficient of variation (CV) = 8.41%] and jug conditions (CV = 4.73%) were used in the analyses. The RFD (CV = 4.49%) was extracted from the same force curve in the jug condition and calculated as the rise in force output during the first 200 ms (RFD_200_) from the onset of the contraction (Levernier and Laffaye, 2019a,b). The onset was determined manually as the point when the force rose with more than 5 N over a 5 ms window after keeping a steady baseline for 1,000 ms (Andersen and Aagaard, 2006; Levernier and Laffaye, 2019a,b). The same researcher performed all the tests and analyses to avoid inter-subject variability.

**Figure 3:**
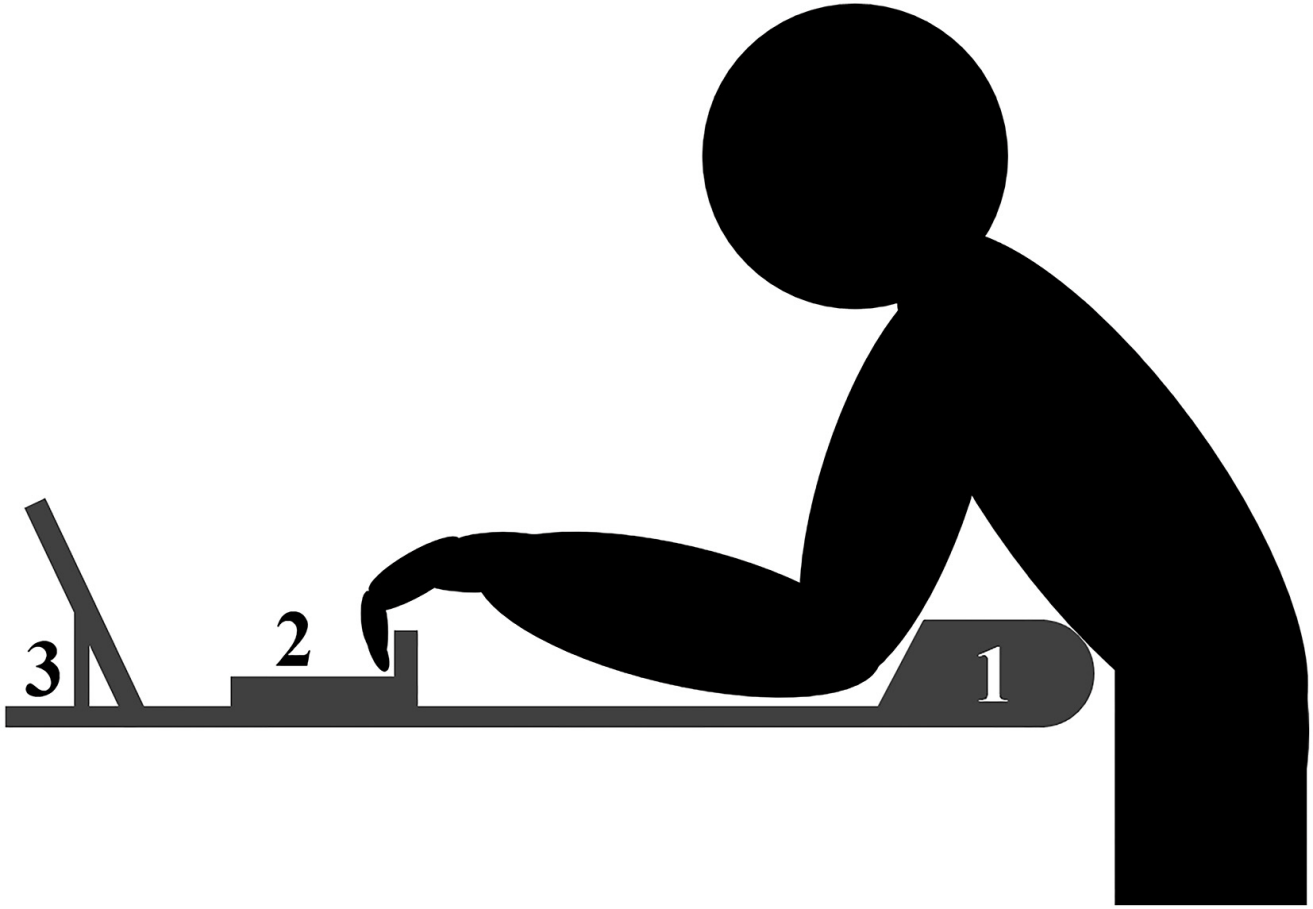
Schematic presentation of the maximal finger-grip strength and intermittent forearm endurance test set-up showing (1) the constraining of the elbow, (2) the fingerboard, and (3) the screen providing real-time feedback of the force.

After performing the pull-ups, maximal isolated half crimp grip force (F_crimp_) was measured while leaning over a table with the elbows fixed in a 90° angle and the arms fully adducted (Figure 3). The elbows were constrained to avoid inclusion of the back and shoulder muscles and only allow the distal phalanges to reach over the rung. The distance between the constrain and the rung was adjustable and was registered to the closest 0.5 mm to allow for identical conditions at pre- and post-test. Subjects held on to the 23 mm rung on a custom fingerboard (Climbro, Innovative Hangboards, Sofia, Bulgaria) with built-in force sensors mounted to the table, using a half crimp grip with a passive thumb. On verbal command, they pulled as hard as they could with the fingers and maintained the maximal force for 3–5 s. One attempt was given, and the highest registered force output was used in the analyses.

After resting for 3–5 min, intermittent forearm endurance was assessed in the same test set-up as the F_crimp_, using 60% of F_crimp_ as the threshold (Balas et al., 2016). The contractions were held for 7 s separated by 3 s rest intervals, and a test was terminated if the force dropped below the 60% threshold+ for more than 1 s (Medernach et al., 2015). The fingerboard registered the force output and real-time visual feedback was provided *via* a screen to allow subjects to adjust their force output. The total work-time (i.e., not including rest-periods) was analyzed. Due to the high level of fatigue and very low technical demands, one attempt was given in this test.

At least 48 h and no more than 5 days after the laboratory tests, climbing performance was assessed on three boulder problems and one lead route. Before testing, a 15-min warm up consisting of light traversing and light-to-moderate bouldering was performed, followed by 10 min of rest. The three boulder problems (A: 6B+, 7 moves; B: 6C, 12 moves; C: 6C+, 6 moves) were performed on a 5-m high wall in a randomized, counter-balanced order. The subjects were given 4 min to work each problem and each problem was separated by 3 min rest. Verbal encouragement was provided, but subjects were never given tips on how to improve their performance. The number of completed moves from the best attempt was recorded and the accumulated score from the three problems was used in the analyses (maximal score = 25). The boulder problems were removed from the indoor wall between pre- and post-test.

After resting for ~15 min, subjects performed the lead climbing performance test. The route was 24 m long on an 18 m high wall and consisted of 66 moves on a progressively steeper wall. The grade of the route was suggested by two independent, highly experienced climbers to be 6c+ (IRCRA 16). Only one attempt was given, and the highpoint (last hold subjects held in a controlled manner) was recorded as a percentage of the total route length. Unexpectedly, two subjects (LCT group) topped the route at both pre- and post-test and were excluded from the analyses. None of the subjects in the BCT group topped the route at pre-test. The subjects were not allowed to practice the route during the intervention.

### Training

As the anecdotal evidence for climbing training currently far outweighs the scientific evidence, the training program was developed in cooperation with highly experienced climbers. The program included a low-to-medium intensity to ensure that the intermediate climbers were able to complete the high-volume training intervention without risk of overuse or acute injuries (Horst, 2016). The subjects were not allowed to perform any climbing or climbing-specific resistance training outside the intervention. They could, however, continue activities such as endurance training and lower-limb resistance training with a low intensity and weekly volume. The researchers had regular contact with the subjects to monitor their training attendance. Both groups trained three times per week for 5 weeks. The BCT group performed two bouldering sessions and one lead climbing session while the LCT group performed two lead climbing sessions and one bouldering session. The session performing the opposite discipline (maintenance-session) was self-regulated by the subjects, but they were instructed to maintain a very low intensity [rating of perceived exertion (RPE) ≤ 3 on a 1–10 scale]. The two primary training sessions consisted of one quality session (RPE <7) emphasizing harder climbs and high effort, and one quantity session (RPE 6) prioritizing a high volume of climbing.

During the 90-min quality session, the BCT group was instructed to perform several quality attempts on a hard boulder and take long rests (3–5 min) between attempts. Subjects were instructed to choose a project that was near their physical limit and maintain an RPE of at least 7. In the 60-min quantitative session, the BCT group completed five sets of four consecutive boulder problems with 5 min rest between sets. The intensity was RPE ≤6.

For the LCT group, the quality session lasted for 1 h with an intensity of RPE 7 or higher. The subjects performed three sets of two consecutive lead climbs with 10 min of rest between sets. A total of six lead climbs were performed in each session. Finally, the 75-min LCT quantity session consisted of completing as many lead climbs as possible within 1 h. Short rests (≥3 min) were allowed between attempts and the intensity was RPE 3 to 6 depending on fatigue levels.

### Statistical Analysis

A Kolmogorov-Smirnov test revealed no deviations from normality in the dataset (*p* = 0.117–0.200). SPSS statistical software (V.25, SPSS Inc., Chicago, IL, USA) was used for the statistical analyses. A mixed-model repeated measures analysis of variance (ANOVA) was used to identify potential differences between the groups. When a group × time interaction or main effect was revealed, Bonferroni *post-hoc* tests were applied to detect the differences. If a main affect for time was revealed, the Bonferroni *post-hoc* was applied to identify the within-groups changes. The results are presented as means ±95% confidence intervals (95% CI) with Cohen's d effect size (ES). Cohen's d ES was calculated as the mean pre-post difference divided by the pooled standard deviation of the change scores and were interpreted as follows: <0.2 = trivial; 0.2–0.5 = small; 0.5–0.8 = medium; >0.8 = large. Statistical significance was accepted at *P* < 0.05 (Cohen, 1988).

## Results

None of the anthropometric or performance-related variables were different between the groups at pre-test (*P* = 0.099–0.990).

The analyses revealed of lead- and boulder climbing performance revealed no group × time interactions (*F* = 1.768, *P* = 0.208 and *F* = 0.079, *P* = 0.784), nor main effects for time (*F* = 1.949, *P* = 0.188 and *F* = 1.717, *P* = 0.215) or group (*F* = 2.127, *P* = 0.170 and *F* = 0.050, *P* = *P* = 0.784; Table 2). Intermittent forearm endurance showed no group × time interaction (*F* = 4.227, *P* = 0.062) or main effect for group (*F* = 0.039, *P* = 0.848), but a main effect for time was found (*F* = 8.061, *P* = 0.015). *Post-hoc* tests showed that intermittent forearm endurance remained unchanged from pre- to post-test in the BCT group (*P* > 1.000), while the LCT group improved by 25 s (*P* = 0.014, 95% CI = 9.7–40.3, ES = 0.55, Table 2). Finally, the analyses of F_crimp_ revealed no group × time interaction (*F* = 0.145, *P* = 0.710) or main effect for group (*F* = 0.398, *P* = 0.540), but did reveal a main effect for time (*F* = 8.157, *P* = 0.014). Further analyses showed that F_crimp_ was improved by 48 N following BCT (*P* = 0.030, 95% CI = 13–82), but not LCT (*P* = 0.416; Table 2).

**Table 2:** The pre- and post-results for the two groups with Cohens d effect size (ES) for the change.

	**Lead climbing training group**			**Boulder climbing training group**		
	**PRE**	**POST**	**ES**	**PRE**	**POST**	**ES**
Boulder (*n*)	14.4 (3.84)	15.1 (1.8)	0.21	14.6 (3.21)	15.8 (2.1)	0.34
Lead (%)	61.0 (23.2)	61.2 (24.1)	0.01	36.4 (9.9)	44.6 (18.9)	0.42
Intermittent (s)	132 (93)	157 (48)^*^	0.55	138 (42)	142 (36)	0.10
F_crimp_ (N)	803 (93)	839 (106)	0.34	751 (153)	799 (154)^*^	0.35
Rung F_avg_ (N)	260 (51)	285 (183)	0.23	259 (109)	323 (152)	0.41
Jug F_avg_ (N)	401 (130)	451 (146)^*^	0.41	392 (153)	458 (198)^*^	0.31

* *Significantly different from pre-test (P <0.05)*.

*Values are presented as mean (95% CI)*.

*Note that two participants successfully completed the lead climbing route and were excluded from this analysis (n = 6 in each group)*.

*The boulder performance is presented as number of completed moves (n), while the lead climbing performance is presented as percentage of the route climbed (%). Results from the intermittent forearm endurance is gives in seconds (s), and force (F_avg_) in the rung, jug and crimp (F_crimp_) conditions are given in Newton (N)*.

**Figure 4:**
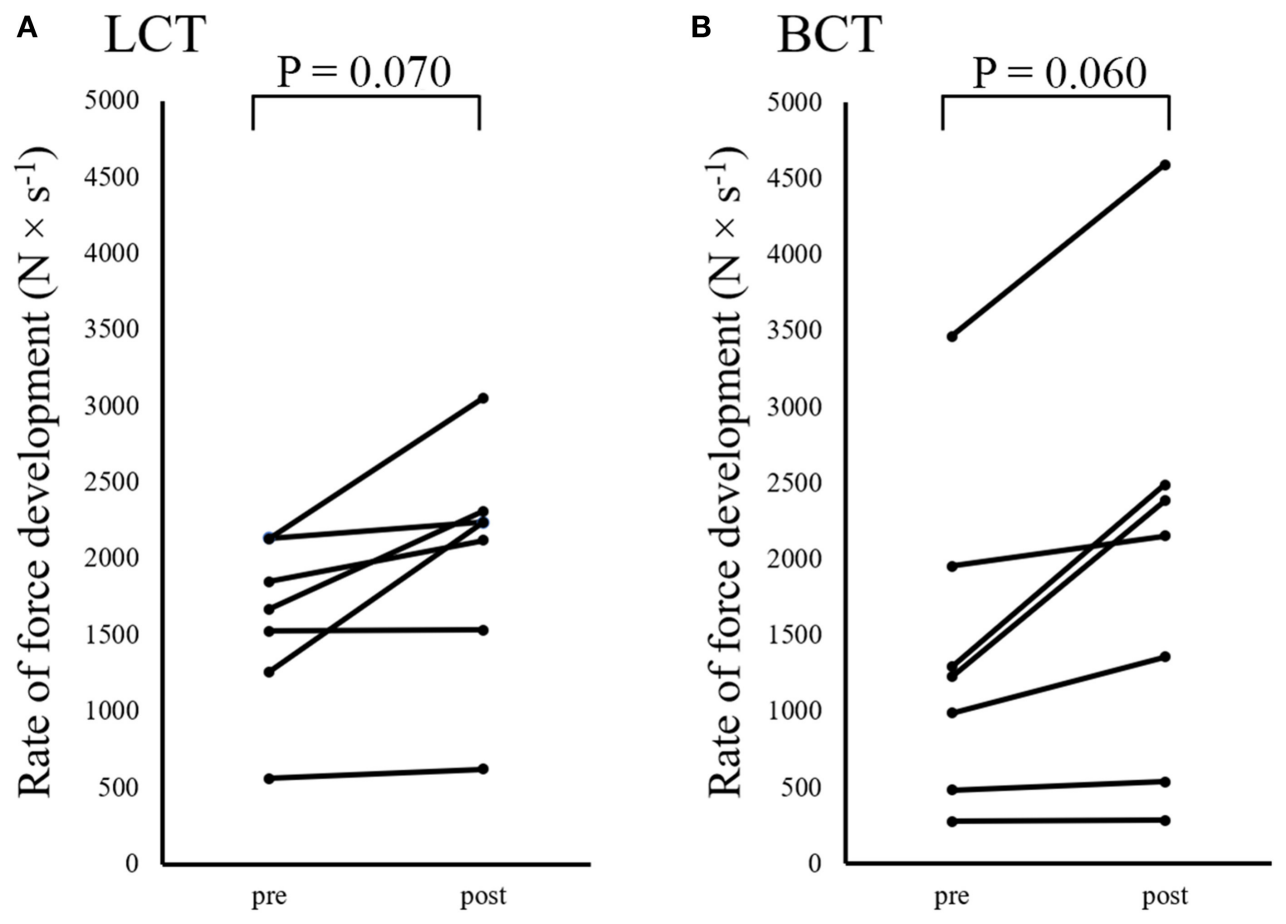
Individual pre- to post-test changes in the rate of force development (RFD) test on the jug holds for **(A)** the lead climbing training group (LCT) and **(B)** the boulder climbing training group (BCT).

The analyses of F_avg_ in the rung and jug conditions showed no group × time interactions (*F* = 2.662, *P* = 0.129 and *F* = 0.347, *P* = 0.567), nor main effects for group (*F* = 0.072, *P* = 0.793 and *F* < 0.001, *P* = 0.992). However, a main effect for time was found for both conditions (*F* = 13.605, *P* = 0.003 and *F* = 17.539, *P* = 0.001). Further analyses showed that F_avg_ in the jug condition improved by 49.7 N for the LCT group (*P* = 0.046, 95% CI = 9.7 N−89.7 N), and by 66.0 N for the BCT group (*P* = 0.049, 95% CI = 11.5 N−120.5 N). Contrarily, F_avg_ in the rung condition did not improve for either the LCT (*P* = 0.084, 24.9 N, 95% CI = 1.2–48.5 N) or the BCT group (*P* = 0.054, 64.3 N, 95% CI = 10.1–118.5 N; Table 2). Regarding RFD, no group × time interaction (*F* = 0.377, *P* = 0.551), or main effects for group (*F* = 0.056, *P* = 0.817) was detected, but there was a main effect for time (*F* = 15.196, *P* = 0.002). Further analyses showed that neither group significantly improved RFD (Figure 4).

## Discussion

This study compared the effects of prioritizing lead climbing or boulder climbing for 5 weeks. Despite no changes in climbing performance, both training groups improved climbing-specific strength as assessed during isometric pull-ups using the jug holds. In accordance with the prioritized discipline (Fanchini et al., 2013; Stien et al., 2019), only the LCT group increased intermittent forearm endurance, whereas only the BCT group improved isolated finger-grip strength. In disagreement with the hypotheses and acute studies that have identified differences in climbing-specific strength and endurance between athletes specializing in lead- or boulder climbing (Fanchini et al., 2013; Laffaye et al., 2014; Fryer et al., 2017; Stien et al., 2019; Levernier et al., 2020), the changes in the tested variables were not statistically different between the two groups. The findings could be attributed to the relatively short training period, low intensity, or the small changes to the subjects' regular training volume. However, the aim of the study was to examine the effects of prioritizing one discipline while maintaining a high ecological validity. Making considerable changes in other factors (e.g., climbing volume) would have confounded the findings. The study population had considerable climbing experience (~5–8 years), but at an amateur level. Due to the intermediate-to-advanced performance level, one could have expected more rapid and distinct changes. Unfortunately, the current results add to the body of literature not being able to demonstrate differences between training modalities in improving climbing performance (Hermans et al., 2017; Philippe et al., 2019) or performance in climbing-specific tests (López-Rivera and González-Badillo, 2012, 2019). Taken together, these findings highlight the need for interventions with higher intensities and longer durations in prospective climbing research. Future studies should also consider monitoring the climbing volume more directly (i.e., vertical meters climbed and number of moves).

Both the LCT (ES = 0.41) and BCT groups (ES = 0.31) meaningfully improved F_avg_ in the jug condition. However, and in contrast with the hypotheses, the improvement was not significantly different between the groups. Moreover, RFD_200_ and F_avg_ in both the rung condition did not improve in any of the groups. Previous investigations have reported RFD and F_avg_ being discriminatory factors between lead- and boulder-climbers (Fanchini et al., 2013; Fryer et al., 2017; Stien et al., 2019), likely due to the higher intensity and more explosive nature of bouldering (White and Olsen, 2010; Fanchini et al., 2013; Laffaye et al., 2014). However, and as previous studies have speculated (Fanchini et al., 2013; Stien et al., 2019), the observed differences between climbing disciplines could be the result of climbers choosing to prioritize one discipline based on their inherent abilities, rather than of the physiological adaptations specific to the discipline. This adds to the difficulty of the present investigation to detect distinct differences after only a 5-week training period. Moreover, as the studies included more accomplished climbers than the current investigation, their discipline-specific attributes may have been more distinct. The current findings are in line with those of Philippe et al. (2019) who reported similar improvements in two groups who participated in either a lead- (muscular endurance) or boulder climbing-specific (muscular hypertrophy) training program. Despite not reaching statistical significance it should be noted that the observed effect sizes for the F_avg_ in the rung and jug conditions might reflect different adaptations to the two training interventions. For example, the LCT group displayed effect sizes of 0.23 (16.2%) and 0.41 (31.8%) in the rung and jug conditions, respectively, while the BCT group achieved effect sizes of 0.41 (28.7%) and 0.31 (18.9%). Whereas, increased isometric pull-up strength might have been mostly mediated by increased pulling-apparatus strength for the LCT group, the finger strength could have been a more important factor for the BCT group. Importantly, one should consider the low sample size when interpreting the results. Researchers may consider these findings when designing prospective studies aimed at identifying possible differences between the two disciplines and strive to include a higher number of participants.

Although the change in forearm endurance was not significantly different between groups, only the LCT group achieved an improvement in this parameter. Moreover, only the BCT group demonstrated improved F_crimp_. Whereas the short, steep and explosive nature of boulder problems might represent a highly strength-specific training stimulus (White and Olsen, 2010; Garber et al., 2011; Fanchini et al., 2013), the longer duration maintaining a lower average intensity during lead climbing is likely a more specific training stimulus for the intermittent forearm endurance test (Sale and MacDougall, 1981). Importantly, the threshold for the intermittent forearm endurance test was calculated at pre- and post-test to maintain an identical relative intensity (i.e., 60% of pre-F_crimp_ at pre-test and 60% of post-F_crimp_ at post-test). Since an improvement in the F_crimp_ was observed in the BCT group, one may speculate that an increase in the threshold force at post-test would reduce potential improvements in forearm endurance. However, since the aim of the forearm endurance test was to assess the capability of maintaining force at a given relative intensity (60% of F_crimp_), using the same absolute force threshold at post-test would render the pre- and post-tests incomparable. Finally, it is possible that using a threshold lower than 60% of maximal force, thereby allowing for a longer test duration, could have favored the LCT group. This could have resulted in a significant difference between the training groups due to the higher specificity of lead climbing with regards to climb duration (i.e., 120–420 s for lead climbing competitions and only around 30 s for bouldering) (White and Olsen, 2010).

This study adds to the scarce body of interventional studies unable to produce distinct differences between lead- or boulder climbing, or resistance training specific to the two disciplines. Hence, it could be speculated that longer periods prioritizing one discipline is needed, whereas an intervention of limited duration may not be sufficient to cause distinctive differences between groups. Moreover, the fact that some athletes perform on a world class level in both disciplines could indicate that the two disciplines do not represent as different demands as previously assumed (Philippe et al., 2019). Indeed, hard lead climbing undoubtedly requires high levels of finger- and pulling-apparatus strength (as demonstrated by the fact that more accomplished lead climbers are stronger than lower-level lead climbers) (Fryer et al., 2015; Levernier and Laffaye, 2019b), and it is reasonable to assume that bouldering performance is influenced by forearm endurance, considering an attempt in competition style bouldering often lasts around 30 s (White and Olsen, 2010).

The present is one of very few studies (Hermans et al., 2017; Philippe et al., 2019) examining actual climbing performance following climbing-specific training, whereas most of the literature have only assessed strength in climbing-related exercises (López-Rivera and González-Badillo, 2012, 2019; Medernach et al., 2015; Saeterbakken et al., 2018; Levernier and Laffaye, 2019a). Both groups maintained climbing ability in both disciplines and improved most of the tested variables despite having the same weekly climbing training frequency as before the intervention. Hence, the observed improvements in climbing-specific strength could result from the changes in the structure of their climbing sessions. Importantly, despite emphasizing one discipline and only performing one weekly low intensity (RPE = 3) maintenance-session of the other, no reduction in performance was observed in either discipline. The results suggest that specific climbing training can increase climbing-specific strength, but increased strength may not be directly associated with improved climbing performance. These findings have implications for researchers designing studies examining the effects of climbing-specific strength training, suggesting that climbing performance should be examined alongside climbing-specific strength and endurance. It should be noted that the lead climbing test used in the present study may not have been suited for identifying changes among the included climbers as several subjects reported falling due to other factors (e.g., difficulties clipping quickdraws or fear of climbing past the last clipped quickdraw in difficult terrain). Following the low-intensity (i.e., easy lead climbing) training in this study, it is likely that the climbers did not achieve improvements specific to difficult lead-climbing. To more directly examine physiological adaptations such as endurance, it may be preferable to perform lead climbing testing using an auto-belay or tread-wall to exclude psychological factors (Gajdošík et al., 2020). However, climbing past the last clipped quickdraw in difficult terrain is an important ability in lead-climbing, suggesting that future research should include high-intensity climbing training to improve performance in demanding climbing-situations.

Although the present study provides novel insight into the effects of climbing training on climbing performance and performance in climbing-specific tests, the study has some potential limitations that should be considered when interpreting the results. First, intermediate and advanced climbers were included in the study and the findings may therefore not be generalizable to elite climbers. Another potential limitation was the heterogeneity of the included participants at baseline (see Figure 4) which challenge the ability to detect significant differences with the relatively low study-sample. Moreover, the relatively short intervention period, low training intensity and small study sample could challenge the statistical power of the study. Future studies may be able to detect differences between groups using a larger population or a longer intervention period. The fact that no familiarization was performed prior to the experimental session could be viewed as a limitation. However, the three attempts were consistent (CV = 4.49–8.41%), and the tests required very low technical performance. Moreover, the subjects performed both disciplines, which could reduce the potential between-groups differences. Still, as the included subjects conducted both lead- and boulder-climbing before the study, excluding one discipline in the intervention period could produce differences between the groups through a decreased performance in that discipline. Finally, although the intensity and duration of the sessions were regulated, the climbing style (e.g., hold types and steepness) were self-selected. Hence, inter-individual differences in climbing style may have influenced the results.

## Practical Applications

This study was one of the first to examine the effects of climbing training on climbing performance, whereas many previous investigations have focused only on climbing-specific strength or endurance training on performance in climbing-specific tests. Interestingly, meaningful improvements were observed for many of the measured strength and endurance variables while climbing performance remained unchanged. Researchers should be aware of these findings when designing future interventional studies and consider including climbing performance in the testing. Further, block periodization could be a viable training method in complex sports such as rock climbing which require development of different properties (e.g., muscular endurance and explosiveness) (Issurin, 2016). The current findings suggest that block periodization in rock climbing (i.e., prioritizing most of the training within a 5-week period on one discipline) can be safely implemented among intermediate and advanced climbers without risking a declined in performance in the other discipline. One very low volume and intensity (RPE ≤3) session performing the other discipline may be sufficient for maintaining performance. Of note, the BCT group achieved larger ES for the change in both lead climbing and bouldering performance, suggesting that a longer bouldering training period could result in superior improvements in both disciplines in this group of climbers. Future research should examine the effects of periodized vs. non-periodized climbing or climbing-specific training.

## Conclusions

In conclusion, no significant differences were produced between the groups in either climbing performance nor climbing specific strength- and endurance tests after the intervention period. However, 5 weeks of performing either mainly lead or boulder climbing improved pull-up strength among intermediate-to-advanced climbers with considerable climbing experience. Although not significantly different between the groups, a 5-week structured lead climbing training regime significantly improved intermittent forearm endurance, whereas only boulder climbing training improved isolated finger strength. Future research is needed to identify whether similar effects occur among elite climbers.

## Data Availability Statement

The original contributions presented in the study are included in the article/Supplementary Materials, further inquiries can be directed to the corresponding author.

## Ethics Statement

The studies involving human participants were reviewed and approved by Norwegian Centre for Research Data. The patients/participants provided their written informed consent to participate in this study.

## Author Contributions

NS, TF, EH, and VV were responsible for the data collection. NS, VA, and AS performed the data curation and statistical analyses. NS drafted the article, while all remaining authors critically revised the draft. All authors contributed to the design and conceptualization of the study.

## Conflict of Interest

The authors declare that the research was conducted in the absence of any commercial or financial relationships that could be construed as a potential conflict of interest.
